# Supplementary material for: A Global Screen for Assembly State Changes of the Mitotic Proteome by SEC-SWATH-MS
Source: Cell Syst. 2020 Feb 26;10(2):133–155.e6. doi: 10.1016/j.cels.2020.01.001 (PMC7042714; doi:10.1016/j.cels.2020.01.001)

Q9P2K2 | TXD16_HUMAN | TXNDC16 ERP90 KIAA1344
Monomer MW [kDa]: 93.572 Monomer expected elution fraction: 41

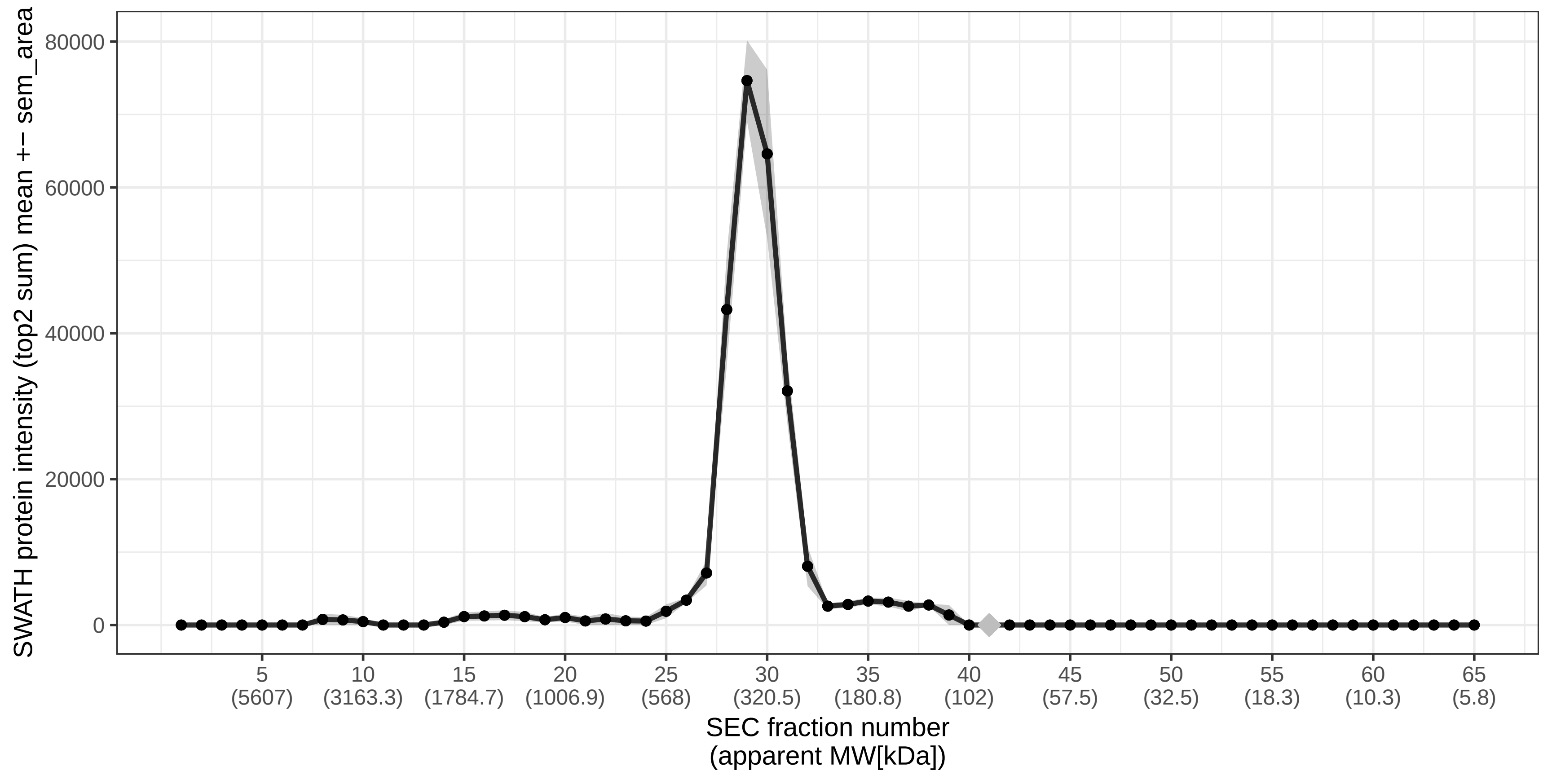

Supplement: Data S1. SEC-SWATH-MS Protein Chromatograms, Related to Figure 1 [file mmc6.zip › SECchrom_Q9P2K2_TXD16_HUMAN_TXNDC16_ERP90_KIAA1344.pdf]
